# Development of a Line of Care for the Health of People Who Engage in Chemsex: Protocol for a Multimethod Study

**DOI:** 10.2196/84068

**Published:** 2026-03-26

**Authors:** Lariane Angel Cepas, Isadora Silva de Carvalho, Alvaro Francisco Lopes de Sousa, Caíque Jordan Nunes Ribeiro, Shirley Verônica Melo Almeida Lima, Anderson Reis de Sousa, Carlos Arterio Sorgi, Ricardo Nakamura, Sumaé Embaló, Ivone Aparecida de Paula, Naila Janilde Seabra Santos, Rodrigo Pierre de Freitas, João Geraldo da Silva Netto, Talita Morais Fernandes, Ana Paula Morais Fernandes

**Affiliations:** 1 Department of General and Specialized Nursing Ribeirão Preto School of Nursing University of São Paulo Ribeirão Preto, SP Brazil; 2 Campus de Três Lagoas Universidade Federal de Mato Grosso do Sul Três Lagoas, Mato Grosso do Sul Brazil; 3 Public Health Research Centre Comprehensive Health Research Center (CHRC), REAL NOVA University Lisbon Lisbon Portugal; 4 Department of Nursing Federal University of Sergipe Aracaju, Sergipe Brazil; 5 School of Nursing Federal University of Bahia Salvador, Bahia Brazil; 6 Department of Chemistry Ribeirão Preto Faculty of Philosophy, Science, and Letters University of São Paulo Ribeirão Preto, SP Brazil; 7 Department of Computer Engineering and Digital Systems Polytechnic School University of São Paulo São Paulo, SP Brazil; 8 Institute of Mathematics, Statistics and Computer Science (IME) University of São Paulo São Paulo, SP Brazil; 9 São Paulo State Health Department Reference and Training Center for STDs/AIDS São Paulo, SP Brazil; 10 Multiverso Institute São Paulo Brazil; 11 Higher Institute of Lisbon and Tagus Valley Lisbon Portugal

**Keywords:** Brazil, chemsex, harm reduction, Health Care Network, HIV prevention, Knowledge-to-Action framework, line of care, men who have sex with men, public health, sexualized drug use

## Abstract

**Background:**

Chemsex, defined as the intentional use of psychoactive substances to enhance sexual experiences, is associated with increased risk of sexually transmitted infections (STIs), mental health harms, and disruptions in continuity of care, particularly among men who have sex with men. In Brazil, health services lack an integrated, stigma-sensitive care pathway (CP) for prevention, clinical management, and harm reduction. Embedding a CP within the Health Care Network (HCN) is essential to organize access, continuity, and quality of care.

**Objective:**

This study aims to develop an evidence-informed CP for people who engage in chemsex, grounded in the needs of users, managers, and health professionals within the HCN, and supported by educational tools and implementation strategies.

**Methods:**

This sequential multimethod study is guided by the Knowledge-to-Action framework. Phase 1 (predevelopment) focuses on establishing partnerships and mapping the local context. Phase 2 (Knowledge Creation) includes (1) cross-sectional surveys with adults from the general population (target n≈1600) to estimate chemsex prevalence and associated factors, and with managers (n≈54) and health professionals (n≈135) to assess knowledge, attitudes, practices, barriers, and stigma; (2) a scoping review following established methodological guidelines; and (3) triangulation and concept mapping to integrate quantitative and qualitative findings. Data will be collected via REDCap (Research Electronic Data Capture), online and in person, during testing events. Quantitative analyses will involve descriptive statistics and regression models. Interviews and focus groups will undergo thematic analysis supported by Nvivo software. Educational products will be developed and evaluated for appearance, content, and usability by experts using a content validity index ≥0.78 as a cutoff. Phase 3 (Action Cycle) includes adapting knowledge to the local context, co-designing and validating the CP with stakeholders, piloting implementation in priority municipalities, and establishing monitoring processes. Process indicators (eg, number of trained professionals, educational activities, rapid tests performed, and app engagement) and outcome indicators (eg, STI testing and linkage to care, service use across the HCN) will be monitored through official information systems.

**Results:**

Ethical approval was obtained in April 2025. Recruitment and data collection began in June 2025 through online and in-person strategies. By December 2025, 3061 individuals had been screened online, and 1723 participants had undergone rapid testing for STIs. Data collection is expected to conclude by June 2026, followed by data cleaning and preliminary analyses between April and June 2026, inferential and qualitative analyses between July and September 2026, and CP development and validation between October and December 2026.

**Conclusions:**

This protocol will generate a CP tailored to chemsex and validated educational resources to support harm reduction, mental health, and STI prevention within the HCN. Findings are expected to inform inclusive policies, reduce stigma, and strengthen care coordination within Brazil’s Unified Health System.

**International Registered Report Identifier (IRRID):**

DERR1-10.2196/84068

## Introduction

### Overview

The term chemsex refers to the intentional use of psychoactive substances with the aim of intensifying and/or prolonging sexual experiences [1]. Although documented in diverse populations, the phenomenon is particularly frequent among men who have sex with men (MSM) [2], with elevated and heterogeneous prevalence rates reported in Brazil [3]. Review studies [4] indicate that, among MSM samples, chemsex prevalence ranges from 27% to 69.9%, varying according to substance profiles and recruitment contexts [5]. These data position chemsex as a public health challenge and justify the development of contextually informed and socially grounded care responses.

At the international level, reviews [6,7] underscore that the specific mix of drugs involved in chemsex is not static but depends on local scenes and markets. Nonetheless, crystal methamphetamine, gamma-hydroxybutyrate (GHB), gamma-butyrolactone (GBL), and mephedrone constitute the core most frequently described in European studies [7]. These patterns are associated with high-risk sexual behaviors [8] (eg, condomless sex with multiple partners and prolonged encounters), a higher burden of sexually transmitted infections (STIs) [7,8], and cooccurrence with events such as the mpox outbreak [9], as well as mental health harms, anxiety, depression, substance-induced psychotic symptoms, and social vulnerabilities [10,11].

In Brazil, understanding chemsex requires attention to specific market dynamics and local cultures of use. In addition to the “classic” trio described in European settings, cocaine powder, crack, ketamine, 3-, 4-methylenedioxymethamphetamine, cannabinoids, poppers (alkyl nitrites), and the adjuvant use of pharmaceuticals such as sildenafil/tadalafil, often combined with alcohol and other substances in party circuits and app-mediated encounters, are relevant [12,13]. Brazilian studies point to widespread availability and use of cocaine/crack and polysubstance use patterns that shape the sexualization of substance use among urban segments, including MSM, but not limited to them [5,14,15]. This drug ecology, which is more diverse than that depicted in the “classic” chemsex literature, reinforces the need for care pathways (CPs) that integrate harm reduction, mental health, and clinical emergency management (eg, GHB/GBL intoxication and stimulant-associated cardiovascular events).

It is also important to recognize that the sexualization of substance use is not limited to MSM. Studies have estimated the pooled prevalence of sexualized substance use (SSU) and chemsex in the general population at around 19.9%, with variations by sex and context, suggesting that sexualized use practices occur across different groups and settings [16]. Although Brazil lacks robust population-based estimates for SSU, the international evidence broadens the scope of surveillance and planning of interventions, including for populations not classified as “key” yet potentially exposed to biomedical and psychosocial risks.

From a service perspective, the research problem arises from the convergence of three gaps: (1) informational—fragmented data on prevalence, polysubstance use patterns, and therapeutic itineraries; (2) care-related—the absence of standardized screening and care flows for early detection, clinical/psychosocial management, and continuous linkage; and (3) organizational—limited integration across points of care (primary care, specialized services, mental health, emergency care, and harm reduction), with consequences for continuity and coordination of care [17,18]. These gaps are exacerbated by stigma and perceived confidentiality barriers, which inhibit disclosure of use and hinder engagement [5,7,10]. In parallel, the intervention literature suggests that multicomponent strategies, such as structured screening, brief counseling, peer educators, digital technologies, and care navigation, are promising for reducing harms and improving outcomes [19].

Brazil has a normative framework capable of guiding integrated responses through the Unified Health System (SUS)/Health Care Network (HCN), established to overcome care fragmentation and organize thematic CPs (eg, HIV/AIDS, viral hepatitis, STIs, mental health, psychosocial care, and harm reduction), with primary health care serving as the coordinator of care [20]. Within this framework, the articulation between surveillance and care, including through regional surveillance groups and bodies, is designed to ensure timely access, longitudinality, comprehensiveness, and effective communication among network points [21]. Despite this, specific CPs for chemsex have not yet been consolidated, creating gaps in STI prevention, management of intoxications and related harms, and the provision of mental health support and psychosocial rehabilitation [19,20].

Thus, the development of a CP for chemsex adapted to the SUS/HCN is justified by (1) high prevalence among MSM and evidence of SSU in the general population; (2) local drug compositions that amplify clinical risks (stimulants, central nervous system depressants, and drug interactions) and psychosocial vulnerabilities; (3) gaps in screening, management, and linkage; and (4) the opportunity to integrate knowledge and practices (clinical care, harm reduction, mental health, testing, and combination STI prevention) into a clear care trajectory with process and outcome indicators [5,17]. Such a CP should include identification tools (screening questions and warning signs), person-centered care packages (including self-testing and linkage), protocols for toxicological emergencies (eg, GHB/GBL), psychosocial support and relapse prevention, and care navigation to reduce losses along the care continuum.

Accordingly, this study aims to develop and pilot-validate a CP for people who engage in chemsex within the SUS/HCN, informed by evidence and sensitive to stigma, articulating surveillance, primary care, specialized services, mental health, and harm-reduction strategies, with clearly defined process and outcome indicators. The research questions guiding this study are as follows: (1) What is the prevalence of chemsex, and what factors are associated with it, in priority municipalities (with an emphasis on MSM and comparisons with other groups)? (2) What gaps, barriers/facilitators, and training needs do managers and professionals identify across HCN care points regarding prevention, testing, toxicological emergency management, mental health, and linkage? (3) Which components and implementation strategies (screening, brief counseling, peer educators, digital tools, navigation) are feasible, acceptable, and scalable in the SUS?

### Objective

To develop a CP for the health of individuals who engage in chemsex, based on the analysis of health needs and the experiences of users, managers, and professionals within the HCN.

## Methods

### Study Design

The study uses a multimethod design with a sequential approach, structured into three main phases: (1) predevelopment, (2) Knowledge Creation, and (3) the Action Cycle, as proposed by the Knowledge-to-Action (KTA) framework [22]. This model, grounded in evidence-informed change theories from the fields of health, social sciences, education, and management, has been widely used to implement health interventions.

The study will be conducted in the state of São Paulo, focusing on both urban and regional contexts. Specifically, the research involves the general population within São Paulo, particularly individuals with an active sexual life, in order to estimate the prevalence of chemsex and identify associated factors. Additionally, the study targets health care professionals and managers working in public health services within the HCN. This includes key personnel from the epidemiological surveillance groups (ESG) across the state, who are responsible for monitoring epidemiological data and coordinating health interventions, including those related to STIs, HIV/AIDS, and other public health concerns. The diverse settings in São Paulo provide a comprehensive context for understanding chemsex practices and the corresponding health care responses at both individual and systemic levels.

### Study Population

#### Study Population, Eligibility, and Recruitment Procedures

The study population will comprise two target groups, aligned with the specific objectives:

1. Individuals from the general population with an active sexual life, to estimate the prevalence of chemsex and identify associated factors.
2. Health managers and health care professionals working in public services linked to the HCN.

#### General Population

##### Inclusion Criteria

Participants aged ≥18 years, residing in Brazil, being sexually active in the past 12 months, and willing to answer sensitive questions regarding sexual behavior, psychoactive substance use, mental health, and access to health care services are eligible for this study. Participation will occur only after written informed consent. For online respondents (REDCap [Research Electronic Data Capture]; Vanderbilt University), consent will be obtained electronically: participants will read the informed consent form (ICF) and indicate agreement by marking the mandatory checkbox before accessing the questionnaire.

For participants recruited in person, the ICF will be presented and signed physically by both the participant and the responsible researcher. In both modalities, consent will be formally documented, and a copy will be provided, digital for online respondents and printed for in-person participants.

##### Exclusion Criteria

Individuals with significant cognitive or emotional impairments that prevent comprehension or completion of the instrument, and those who refuse to participate or decline to sign the ICF, are excluded from participation. Participation is not contingent upon engaging in chemsex; the sample must include individuals who do and do not practice chemsex to enable unbiased prevalence estimates and analysis of associated factors.

#### In-Person Recruitment and Participant Procedures

To recruit participants for the survey, chain-referral sampling (snowball sampling) adapted to the virtual environment will be used, a method commonly used with hard-to-reach populations. In this approach, eligible participants receive the study link and are invited to share it through their social networks and contacts with other individuals who meet the criteria.

In parallel, targeted dissemination will be carried out on social media platforms (Facebook and Instagram). The research team will create a fixed post on the project’s web page containing a brief description of the study and an invitation to participate, including a link to the questionnaire. This post will be boosted (paid promotion) to reach all regions of the state of São Paulo and will remain active until the planned sample size is reached. Considering the higher prevalence of chemsex reported among MSM in the literature, the Hornet platform, popular within this community, will also be used. Hornet’s geolocation features will allow more targeted outreach to users across different regions, enhancing reach. Another key factor is the familiarity and comfort many MSM have with this platform, commonly used for social interaction, which may increase response rates. All recruitment materials will use neutral, nonstigmatizing language, and no personally identifiable information will be collected through dissemination channels.

For in-person questionnaire administration, individuals will be invited to participate voluntarily. After acceptance and signing of the ICF, one-on-one interviews will be conducted in settings that ensure privacy. Confidentiality and anonymity will be guaranteed throughout the entire data collection process, and data will be coded to protect participant identity. After completing the questionnaire, participants will be oriented and referred for rapid testing for HIV, syphilis, and viral hepatitis. Each data collection session, including rapid testing and questionnaire administration, will last approximately 30 minutes, allowing sufficient time to ensure privacy and participant support. During this interaction, participants will also receive counseling and health education on harm reduction and STI/HIV/AIDS prevention.

#### Context and Systematic Recruitment Procedures (General Population)

To recruit participants for the survey, chain-referral sampling (snowball sampling) adapted to the virtual environment will be used, a method commonly used with hard-to-reach populations. In this approach, eligible participants receive the study link and are invited to share it through their social networks and contacts with other individuals who meet the criteria.

In parallel, targeted dissemination will be carried out on social media platforms (Facebook and Instagram). The research team will create a fixed post on the project’s web page containing a brief description of the study and an invitation to participate, including a link to the questionnaire. This post will be boosted (paid promotion) to reach all regions of the state of São Paulo and will remain active until the planned sample size is reached. Considering the higher prevalence of chemsex reported among MSM in the literature, the Hornet platform, popular within this community, will also be used. Hornet’s geolocation features will allow more targeted outreach to users across different regions, enhancing reach. Another key factor is the familiarity and comfort many MSM have with this platform, commonly used for social interaction, which may increase response rates. All recruitment materials will use neutral, nonstigmatizing language, and no personally identifiable information will be collected through dissemination channels.

For in-person questionnaire administration, individuals will be invited to participate voluntarily. After acceptance and signing of the ICF, one-on-one interviews will be conducted in settings that ensure privacy. Confidentiality and anonymity will be guaranteed throughout the entire data collection process, and data will be coded to protect participant identity. After completing the questionnaire, participants will be oriented and referred for rapid testing for HIV, syphilis, and viral hepatitis. Each data collection session, including rapid testing and questionnaire administration, will last approximately 30 minutes, allowing sufficient time to ensure privacy and participant support. During this interaction, participants will also receive counseling and health education on harm reduction and STI/HIV/AIDS prevention.

#### HCN Managers and Professionals

The study will include managers/coordinators and frontline health professionals (nursing, medicine, psychology, social work, pharmacy, peer educators, among others) who are formally affiliated with a public service within the HCN. Coordination will occur with support from the STD/AIDS Reference and Training Center of São Paulo state (CRT-DST/AIDS-SP) during periodic meetings with the 27 STI/AIDS focal points from the ESG of the state of São Paulo, enabling identification and agreement with municipalities interested in participating.

The study will be conducted in the city of São Paulo and in 27 municipalities selected from the ESG. It will include administration of questionnaires to the 27 regional STI/AIDS focal points of the ESG and to 1 STI/AIDS program coordinator from each selected municipality. Additionally, questionnaires will be applied to health professionals in these municipalities across mental health, STI/AIDS care, emergency and urgent care, primary care, and social assistance.

Municipal selection will be based on the highest incidence of HIV or AIDS, allowing the study to focus on areas where prevention efforts are insufficient or where incidence is rising. This approach will enable the investigation of how the knowledge of managers and professionals influences municipal services and the proposition of tailored interventions for this population. By focusing on localities where prevention and care challenges are most critical, the study will provide a robust and targeted analysis of gaps in health policies.

Recruitment will occur through institutional channels (emails and mailing lists from health departments and coordinations). Formal invitations will be sent to managers and professionals to participate voluntarily, including a link to the ICF and the questionnaire. The ICF will be presented online, and participants must accept it to proceed. Once accepted, they will be automatically directed to the questionnaire to be completed individually on the REDCap platform.

### Data Collection Platform and Instrument

The questionnaires, conducted both in person and online, will be hosted on the REDCap platform (project with HTTPS connection, audit trails, and access controls). The main survey is structured into five sections, with skip logic and input validations:

1. Eligibility, introduction, and ICF (study information and electronic consent)
2. Social and demographic characteristics
3. Sexual behaviors and substance use (lifetime and past 12 months)
4. Chemsex practices and harm reduction (frequency, contexts, substance combinations, adverse events, and care-seeking)
5. Well-being (World Health Organization-5 Well-Being Index [WHO-5]) and quality of life (EQ-5D-5L)

To ensure data integrity, REDCap will be configured to allow only one response per IP address and will include mandatory fields and logical checks to reduce missing data and inconsistencies. No direct personal identifiers will be collected; geographic variables will be aggregated to avoid reidentification.

### Instrument Validation and Pretesting

Prior to fieldwork, the questionnaire underwent content and face validation conducted by 5 expert judges (topics: chemsex/SSU, STIs/HIV, harm reduction, mental health, and health service organization). Judges evaluated the relevance, clarity, and comprehensiveness of the items; their suggestions informed revisions to wording, ordering, and response options. Subsequently, a pretest was performed with 10 participants from the target audience to assess comprehension, flow, and completion time, following predefined criteria for adjustments (eg, >10% “don’t know”/missing responses on any item or recurrent comprehension problems).

### Sample Size and Monitoring

For the general population survey, the primary outcome is the prevalence of chemsex in the past 12 months (yes/no). The sample size was calculated to estimate this proportion with a 95% confidence level and an absolute precision (margin of error) of 3 percentage points, adopting the conservative estimate *P*=.50, which maximizes sample size when true prevalence is unknown [23-25]. The standard formula for prevalence studies was applied:



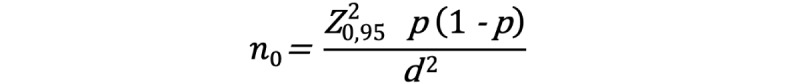



where *Z*_0.95_=1.96, *P*=0.50, *d*=0.03. Thus,



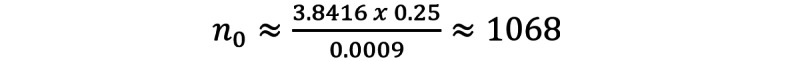



Considering the virtual recruitment method using snowball sampling and targeted campaigns, a design effect (deff) of 1.4 was adopted to accommodate potential intranetwork/region dependence, resulting in n ≈ 1068 × 1.4 ≈ 1495. To account for losses, ineligible cases, and incomplete questionnaires, an additional 10% was added, yielding n≈1645. Based on this justification and logistical constraints, a target sample of approximately 1600 participants was defined, a value that preserves the desired precision and enables stratified analyses by relevant subgroups for the protocol (eg, MSM vs others) and by macroregions [16].

The calculation was verified in G*Power (version 3.1.9.7; Heinrich Heine Universität Düsseldorf), using conservative scenarios (assumed prevalence of 50%, 95% CI, 3-percentage-point precision) and scenarios comparing proportions (MSM vs non-MSM). It is noteworthy that selecting *P*=.50 is a recommended practice when local prevalence is uncertain, as it maximizes sample size and safeguards the planned precision [24,25].

For the module involving managers and health care professionals within the HCN, sampling will be conducted by convenience, operationalized in partnership with the ESG of the state of São Paulo, with targets of ≥3 respondents per service (multiprofessional composition). Although this is not a probabilistic sample, the target of approximately 135 professionals from 5 key health care sectors (mental health, STI/HIV/AIDS, emergency care, primary care, and social assistance) will be included, with a focus on those working with the population who engage in chemsex, that allows descriptive estimates with reasonable precision. The sample for managers will be selected by convenience and will consist of 54 individuals, including 27 regional STI/HIV/AIDS coordinators and 27 additional managers from selected municipalities based on HIV incidence.

### Study Phases

The study is structured according to the KTA framework, a comprehensive model designed to guide the translation of evidence into practice. Although the KTA cycle comprises two overarching and interlinked components (Knowledge Creation and the Action Cycle), this study operationalizes this framework into 3 sequential phases to better organize the development of the Chemsex Care Pathway within Brazil’s HCN. This framework has been widely applied in health, social sciences, and implementation research, drawing on theories of organizational change, knowledge translation, and evidence-based practice [22]. In this protocol, the model is explicitly adapted to support the development, implementation, and evaluation of a CP tailored to chemsex-related needs.

**Figure 1:**
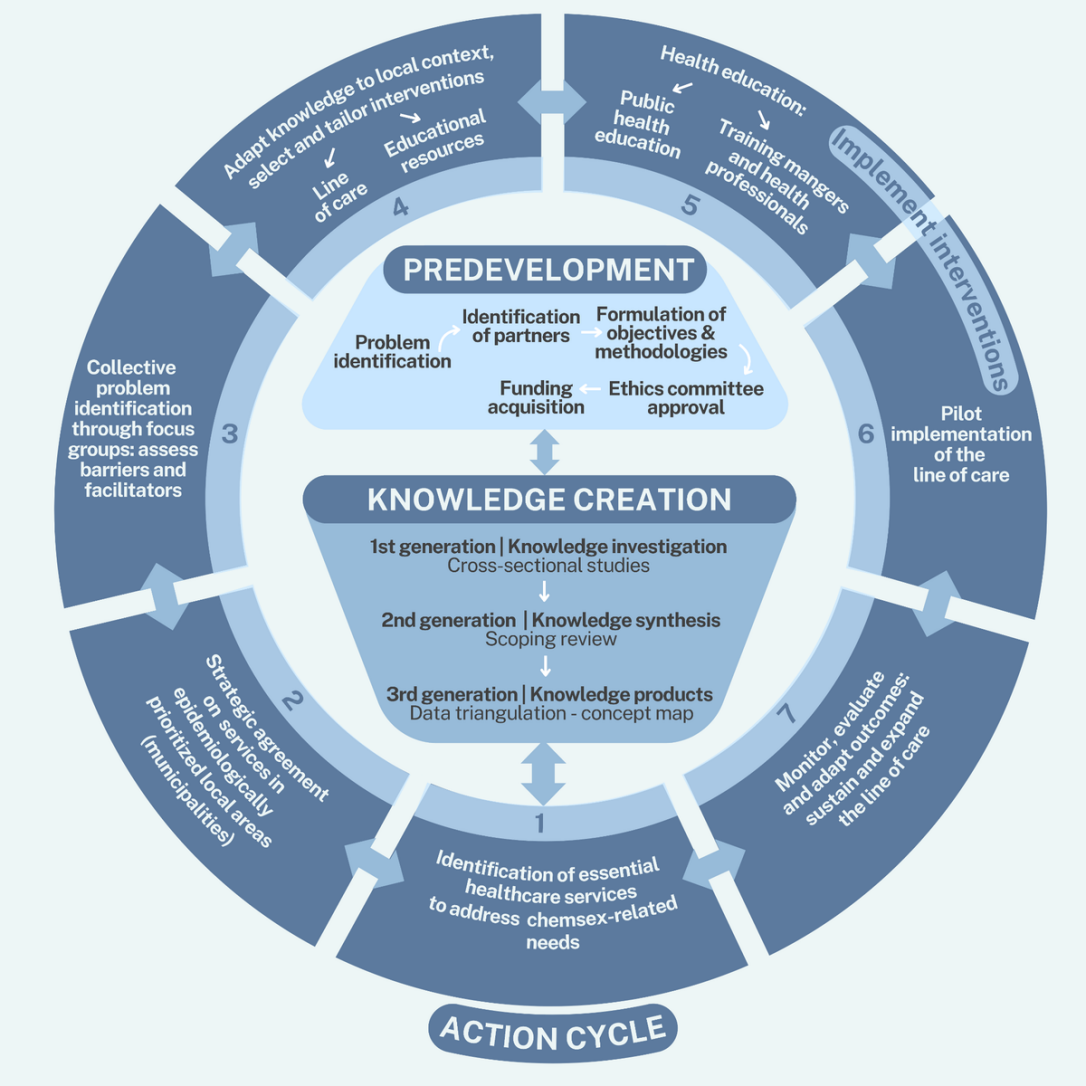
Synoptic overview of the study design adapted from the Knowledge-to-Action framework.

The predevelopment phase lays the groundwork for the study by identifying the problem, engaging stakeholders, and assessing contextual factors relevant to implementation. This preparatory stage ensures that the study is anchored to real-world demands and enables the effective progression of the subsequent phases. The Knowledge Creation phase encompasses primary knowledge investigation, evidence synthesis, and the generation of best-practice tools and products that will inform the CP. Finally, the Action Cycle phase focuses on implementing, monitoring, and sustaining the CP, ensuring that the knowledge generated is systematically contextualized and translated into practical applications within health care settings (Figure 1 [11]).

### Predevelopment

The predevelopment phase establishes the foundational conditions required for successful implementation. In this study, it begins with a comprehensive process of problem identification and contextual assessment, which includes the review of scientific literature, policy documents, and epidemiological indicators (such as STI rates, mental health outcomes, and emergency care use) alongside preliminary consultations with key stakeholders. This process allows the research team to identify system gaps, stigma-related barriers, service fragmentation, and contextual determinants that may influence the feasibility and effectiveness of the intervention.

In parallel, stakeholders (professionals and chemsex users) are systematically mapped and engaged, including municipal and state health authorities, ESG, primary care managers, psychosocial care teams, emergency services, nongovernmental organizations, and community representatives. Their early involvement supports the definition of shared expectations, roles, and resource requirements, thereby strengthening feasibility and fostering joint ownership of the CP.

During this phase, the study also completes ethical preparation and methodological alignment. All study materials (including surveys, interview guides, review protocols, and data collection instruments) are finalized and submitted for ethical approval. This stage ensures coherence between the study’s objectives, methodological approach, and governance structure, thereby establishing a solid basis for the subsequent phases of Knowledge Creation and the Action Cycle.

### Knowledge Creation

The Knowledge Creation phase is structured into 3 progressively refined generations of knowledge production, each designed to address specific methodological requirements of the KTA framework and to provide the level of detail necessary for replication and external evaluation. This phase follows the logic of funneling broad empirical inputs into increasingly targeted and actionable products that will inform the Chemsex Care Pathway.

The first generation (primary knowledge inquiry) consists of a set of methodologically distinct but interrelated cross-sectional investigations targeting 3 key populations: individuals who engage in chemsex, health care managers, and health professionals. For people who engage in chemsex, the survey instrument is designed to capture multiple analytical domains: (1) substance-use characterization, including granular details on substance types, administration routes, polysubstance combinations, temporal sequencing of consumption during sexual encounters, and adverse effects; (2) behavioral and contextual determinants, such as sexual partnering dynamics, motivations, environmental settings, social networks, and indicators of harm (eg, blackouts, nonintentional substance mixing, or emergency care use); (3) biopsychosocial profiling, including mental health indicators, health care use patterns, perceived barriers, and service needs; and (4) biological vulnerability markers, specifically incidence or self-reported history of HIV, STIs, viral hepatitis, syphilis, and mpox.

Surveys for managers and health professionals are structured to address three domains: (1) knowledge, including familiarity with chemsex-related substances, clinical risks, and care protocols; (2) attitudes toward people who engage in chemsex, with measurement of explicit and implicit stigma and discrimination; and (3) practices, including clinical management, referral patterns, institutional barriers, documentation procedures, and training needs. All instruments undergo content validation by experts, followed by cognitive interviews to ensure clarity and construct alignment. Data will be collected via REDCap using both online and in-person recruitment, and statistical analyses will include descriptive modeling, bivariate testing, and regression models to estimate prevalence ratios or odds ratios with 95% CIs.

The second generation (knowledge synthesis) consists of a comprehensive scoping review whose protocol has been published separately as “Healthcare Access Among Individuals Who Practice Chemsex in Brazil: A Scoping Review Protocol” [17]. In accordance with this protocol, the review follows the Joanna Briggs Institute methodology [26-28] and Arksey and O’Malley’s [29] foundational framework. The review question was formulated using the population-concept-context structure, and the search strategy was developed in collaboration with a health sciences librarian to ensure precision and reproducibility. Searches will be conducted in the electronic databases PubMed/MEDLINE, Embase, Scopus, SciELO, LILACS, and PsycINFO, as specified in the published protocol, using free-text terms related to chemsex, sexualized drug use, substance use, and health care access. All retrieved records will undergo dual independent screening at the title/abstract and full-text levels using Covidence, with disagreements resolved by a third reviewer. Data extraction will follow a structured form capturing study characteristics, population details, chemsex-related behaviors, health service use, barriers and facilitators to care, and reported outcomes. Evidence will be synthesized using a mixed methods approach, allowing the integration of quantitative and qualitative findings to comprehensively map existing knowledge. Reporting will follow the PRISMA-ScR (Preferred Reporting Items for Systematic Reviews and Meta-Analyses extension for Scoping Reviews) checklist [30], ensuring transparency in search, screening, extraction, and synthesis procedures. As detailed in the published protocol [17], this methodological structure provides a rigorous and reproducible foundation for understanding health care access among populations who engage in chemsex in Brazil.

The third generation (triangulation and concept mapping) provides the analytical integration needed to translate empirically derived and synthesized evidence into a coherent framework for decision-making. Guided by methodological triangulation principles [31,32], data from the cross-sectional studies, qualitative strands, and the scoping review will be integrated to identify convergence, complementarity, and divergence across sources. A reflexive thematic analysis will be conducted following Braun and Clarke’s [33] established 6-phase framework, as well as their more recent methodological guidance emphasizing reflexivity, analytic transparency, and researcher positionality [34]. The analytic process will include familiarization with the dataset, systematic coding, theme construction, theme review, theme definition, and final reporting. Coding will be performed iteratively by multiple researchers, and regular peer-debriefing meetings will be held to compare coding decisions, refine codebooks, and ensure analytic coherence. Analytic memos and reflexive journals will be maintained throughout to document decision-making processes, positionality considerations, and interpretive influences. To enhance credibility, confirmability, dependability, and transferability, the analysis will incorporate triangulation across stakeholder groups, an audit trail documenting analytic decisions, maintenance of a coding consistency log, and the use of thick descriptions to contextualize findings within the operational realities of the HCN. Following thematic analysis, qualitative themes will be systematically integrated with quantitative findings to identify systemic weaknesses, care discontinuities, risk amplifiers, and context-specific barriers. This integration will support the development of a conceptual map using structured concept mapping approaches [35,36], which will visually articulate the relationships among care needs, service gaps, contextual barriers, and organizational levers for change. The resulting conceptual map will function as a transitional knowledge tool bridging the Knowledge Creation and Action Cycle phases, defining both the theoretical and operational foundations of the Chemsex Care Pathway and guiding the selection of implementation strategies [31,32,35,36].

### Action Cycle

#### Overview

The Action Cycle, as defined within KTA, provides a structured, iterative, and evidence-informed process for translating knowledge into practice and ensuring that interventions are effectively implemented, monitored, and sustained in real-world settings. Conceptually, the KTA model is grounded in well-established theories of change widely applied in health, social sciences, education, and management. These include the Promoting Action on Research Implementation in Health Services framework, which emphasizes the interaction between evidence, context, and facilitation in successful implementation; the Diffusion of Innovations theory, which explains patterns of adoption and spread of new practices; and organizational learning and knowledge use theories, which describe how systems absorb, adapt, and routinize new knowledge [22,37,38].

The Action Cycle begins with a clear identification of the problem, the available evidence, and the contextual factors shaping the local health system. Subsequent stages follow the core steps of the KTA process: adapting knowledge to the local context; assessing barriers and facilitators to knowledge use; selecting and tailoring interventions to meet local needs; implementing evidence-informed strategies; monitoring knowledge use and fidelity; evaluating outcomes and service performance; and sustaining and scaling successful practices. In this protocol, these steps are operationalized through a series of structured activities, including territorial epidemiological assessment, stakeholder agreements, multisectoral focus groups, development and validation of the CP and educational tools, pilot implementation in prioritized municipalities, professional training, and systematic monitoring through national health information systems. Each stage contributes to ensuring that the CP is contextually relevant, institutionally feasible, and capable of improving the quality and continuity of care for individuals who engage in chemsex.

#### Identification of Essential Health Care Services to Address Chemsex-Related Needs

In alignment with KTA, this stage consists of a structured and operationally feasible process for selecting priority municipalities and defining which services within the HCN will participate in the pilot implementation of the Chemsex Care Pathway. Selection will begin with the identification of priority municipalities within the 27 ESG of the state of São Paulo. This process will use routinely available indicators from the Notifiable Diseases Information System, the Viral Hepatitis Information System, and official epidemiological bulletins from the State Health Secretariat. Priority will be given to municipalities with consistently higher incidence of HIV, viral hepatitis, and syphilis over the past 5 years, as well as areas reporting higher demand for STI services, emergency care related to substance intoxication, and psychosocial care needs. This approach relies solely on datasets already used in statewide planning and, therefore, is directly feasible within the project’s scope. Next, a practical territorial assessment will be conducted for each candidate municipality. This includes reviewing the organization of the local HCN (primary care units, specialized STI/HIV clinics, psychosocial care centers, emergency services, and hospital referral units), availability of human resources, and existing care flows documented in municipal or regional planning instruments. These data are routinely compiled by municipal and state managers.

The combination of epidemiological burden and service availability will allow the identification of municipalities with both high need and adequate minimum structure for piloting the CP. This ensures feasibility while maintaining transparency in the selection criteria. Municipalities with clear service gaps but high epidemiological burden may also be included if the local management expresses interest and capacity to participate, reflecting the collaborative nature of implementation within SUS.

**Figure 2:**
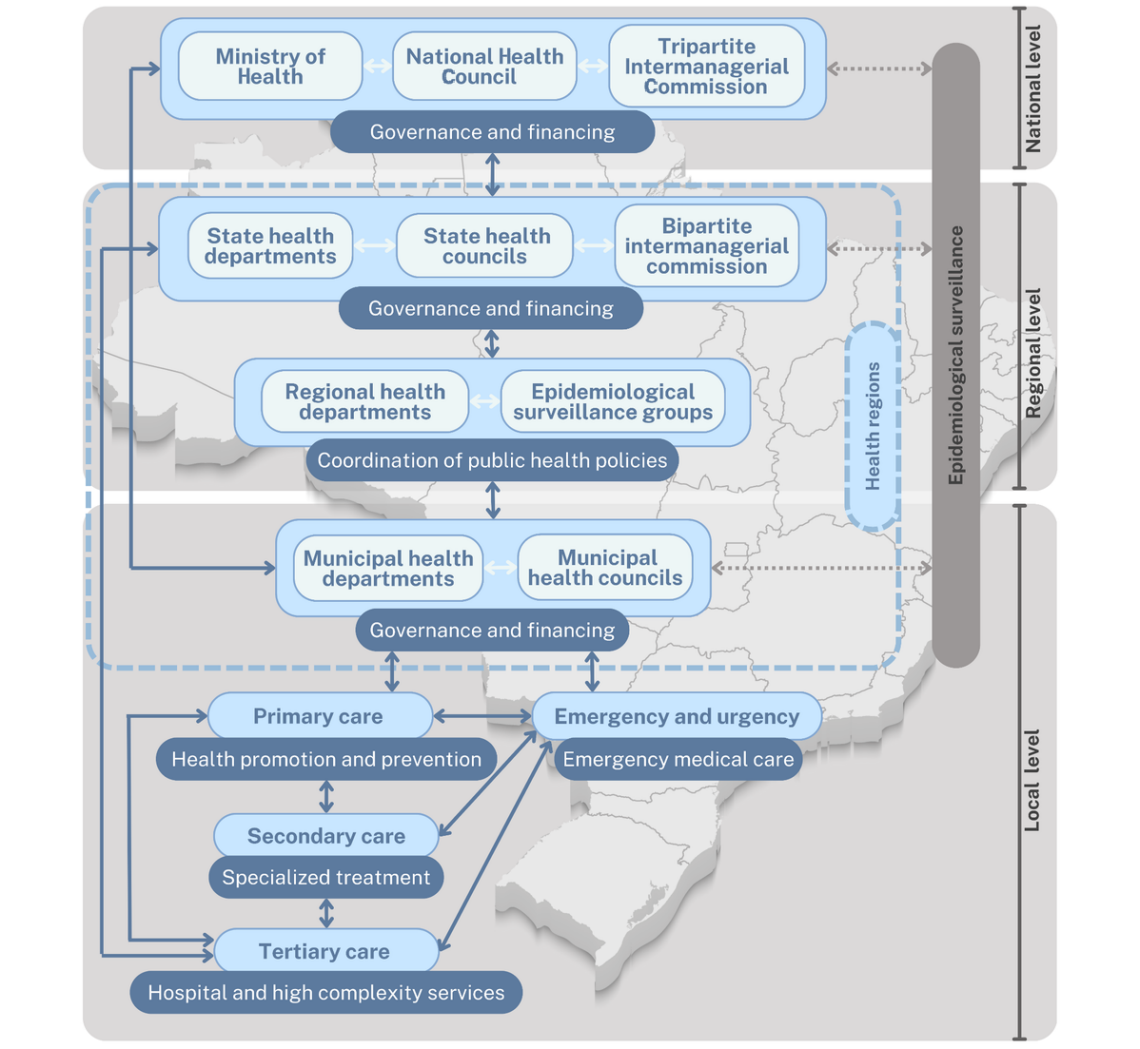
Illustration of the institutional and governance structure of Brazil’s Unified Health System and the Health Care Network, highlighting the coordination across national, state, regional, and municipal levels. This hierarchical organization ensures integrated (national, regional, and local) health care delivery.

Finally, the hierarchical structure of the HCN (comprising primary care, specialized outpatient care, high-complexity care, and emergency/urgent services) will be mapped in each selected territory. This mapping will identify the specific services that will integrate the pathway, their roles within the intervention, and the coordination mechanisms between levels of care. Figure 2 illustrates the governance structure of SUS and its multilevel coordination across federal, state, regional, and municipal spheres, which underpins the organization of the pilot CP.

#### Strategic Agreement on Services in Epidemiologically Prioritized Local Areas (Municipalities)

Following the identification of priority municipalities, formal agreements will be established directly with municipal health managers and the municipal STI program coordinators, in coordination with the regional ESG of the state of São Paulo. This process corresponds to the KTA steps of adapting knowledge to the local context and identifying barriers and facilitators, and is fully aligned with existing SUS planning and governance mechanisms.

These agreements will be carried out through structured virtual or in-person meetings involving the municipal health secretariat, the STI/HIV program coordinator of each municipality, and the respective ESG technical teams. During these meetings, the scope of municipal participation will be defined considering (1) availability and current capacity of STI/HIV rapid testing, (2) organization of primary care teams, (3) presence and functioning of psychosocial care centers, (4) availability and structure of emergency care services, and (5) existing clinical and managerial protocols related to sexual health, substance use, and harm reduction. Because these components are already part of municipal responsibilities, the agreements focus on strengthening and integrating existing service points rather than creating new structures.

Jointly defined operational goals will guide implementation, such as increasing testing availability, expanding harm reduction counseling, improving referral pathways between HCN levels, and formally adopting the Chemsex Care Pathway. A routine monitoring plan will also be established, using feasible process indicators already captured by SUS information systems (eg, number of rapid tests performed, number of professionals trained, or number of referrals completed), ensuring a pragmatic and sustainable approach within municipal workflows.

#### Collective Problem Identification Through Focus Groups: Assess Barriers and Facilitators

This stage uses a structured qualitative approach to ensure that the CP is aligned with the operational realities of the HCN. Focus groups will be conducted with municipal health managers, STI/HIV program coordinators, primary care professionals, psychosocial care providers, emergency care staff, nongovernmental organizations, and individuals who engage in chemsex. Participants will be invited through formal communications issued via municipal health secretariats and the regional ESG, ensuring representation across the main service points implicated in chemsex-related care. Each stakeholder group will participate in 2 sequential focus groups. The first session will present the findings from the primary studies and the preliminary conceptual map, and will use a semistructured analytical matrix to identify operational barriers (such as fragmented referral pathways, insufficient training, limited harm reduction practices, stigma, and gaps in mental health integration) and local facilitators, including existing protocols, experienced teams, or ongoing prevention initiatives. Analytical techniques, including priority ranking and elements of the nominal group method, will be applied to elicit and organize participant perspectives systematically. The second session will focus on the validation of the preliminary CP logic model following the procedures proposed by McLaughlin and Jordan [39], examining the model’s assumptions, internal coherence, feasibility, and alignment with municipal workflows. All sessions will be recorded, transcribed, and subjected to thematic analysis, decision-tracking matrices, and the maintenance of an audit trail. The outputs of this stage will include a refined list of priority problems, a structured assessment of contextual barriers and facilitators, and a stakeholder-validated logic model that will guide subsequent adaptation and implementation steps.

The semistructured interview and focus group topic guides are available as supplementary material (Multimedia Appendices 1 and 2).

#### Health Education

##### Overview

This stage corresponds to the KTA steps of implementing selected interventions and supporting knowledge use through targeted educational strategies directed both at the general population and at health care professionals. The health education component is designed to strengthen awareness, improve risk perception, and promote harm reduction practices among individuals who engage in chemsex, while simultaneously enhancing the technical capacity of professionals across different levels of the HCN.

###### Public Health Education

Public health education will be carried out using a multimodal communication strategy that relies on existing municipal infrastructures and partnerships. Educational content (developed based on the triangulated findings from the primary studies, scoping review, and focus groups) will address risks associated with psychoactive substance use during sexual activity, signs of acute intoxication, safer-use strategies, STI/HIV prevention, mental health warning signs, and recommended pathways for seeking care. Dissemination will leverage municipal health department communication channels, social media platforms, and collaboration with dating applications commonly used by communities (eg, Hornet, Grindr, Scruff, and Tinder), enabling targeted outreach without requiring new technological infrastructures. Partnerships with nongovernmental organizations and community organizations will support community-based educational interventions tailored to diverse and vulnerable populations, including migrants, trans people, sex workers, and individuals with previous experiences of substance use or mental health conditions.

###### Training Managers and Health Professionals

Professional training will be implemented in parallel and will follow a blended learning model that combines in-person workshops with online modules integrated into the continuing education structures already established within the SUS. Training activities will be developed collaboratively with municipal health secretariats and the ESG, ensuring alignment with regional epidemiological profiles and existing service capacities. The educational guide and mobile application developed in Phase 4 will serve as core pedagogical tools, offering standardized, evidence-based content on harm reduction, chemsex-specific clinical management, acute toxicity recognition, STI/HIV prophylaxis pathways, and mental health support strategies. Training sessions will be scheduled to accommodate municipal workflows and will prioritize frontline teams, including primary care professionals, psychosocial staff, urgent and emergency service providers, and specialized STI clinics (ensuring that services most frequently accessed by chemsex users are systematically capacitated). Evaluation of training effectiveness will incorporate pre- and posttraining assessments, feedback forms, and monitoring of knowledge retention and self-reported confidence to apply guidelines in clinical encounters. Together, these population- and professional-directed educational strategies provide the foundation for sustained knowledge use and support the successful implementation of the CP across diverse municipal contexts.

In addition, a structured 20-hour online continuing education course will be developed and made available through digital learning platforms, targeting health professionals at different levels of care (primary care, specialized services, and mental health and STI/HIV programs). This course will include asynchronous modules, recorded lectures, case studies, and practical guidelines, enabling continuous professional development, broad territorial reach, and long-term sustainability of training actions.

##### Pilot Implementation of the CP

###### Overview

The pilot implementation stage corresponds to the KTA steps of applying tailored interventions and supporting the initial use of knowledge within routine service settings. Implementation will take place in municipalities that formalize participation through agreements with local health managers and STI program coordinators, in coordination with the ESG. Instead of requiring new structures, the pilot will embed the Chemsex Care Pathways into the existing municipal service network, including primary care units, specialized STI clinics, psychosocial staff from municipal mental health services, and urgent and emergency care teams. This approach ensures feasibility by relying on already-established HCN infrastructures and workforce capacities. This strategy will allow the CP to address the needs of both urban and semirural contexts, considering the organizational characteristics, service capacity, and epidemiological profile of each local HCN. Implementation will occur in consecutive and integrated phases.

####### Territorial Selection and Situational Diagnosis

Municipalities interested in implementing the CP will be selected based on epidemiological indicators, service infrastructure, institutional commitment, and willingness to adopt harm reduction approaches. A situational diagnosis will be conducted in each site to map available services, care flows, gaps in assistance, and existing intersectoral partnerships.

####### Governance and Institutional Alignment

A formal alliance will be established between CRT-DST/AIDS-SP, local health unit managers, municipal and state health authorities, and key stakeholders. This governance structure will define responsibilities, communication flows, referral protocols, and monitoring mechanisms. Technical committees will be created locally to ensure alignment between policy, management, and care practice.

####### Adaptation of the CP Model to the Local Context

Although based on a standardized care model, the CP will be adapted to the operational, cultural, and territorial realities of each municipality. This includes adjustments in referral flows, integration with existing services (primary care, STI/HIV services, mental health, harm reduction teams, and social care), and tailoring communication strategies to local populations.

####### Training and Capacity Building of Health Teams

Health professionals and managers involved in the CP will receive structured training, including in-person workshops and the 20-hour online continuing education course developed within the project. Training will focus on chemsex, harm reduction, trauma-informed care, sexual health, STI/HIV prevention, and integrated care approaches.

####### Operationalization of Care Flows

The CP will be implemented through structured CPs linking community-based entry points, primary health care, specialized services, mental health services, and social support networks. These pathways will ensure early identification, welcoming, risk assessment, clinical follow-up, harm reduction interventions, access to preexposure prophylaxis/postexposure prophylaxis, STI diagnosis and treatment, psychosocial care, and referral when needed.

####### Monitoring and Evaluation

Continuous monitoring will be conducted using quantitative and qualitative indicators, including service use, user flow, adherence to care, professional performance, and user satisfaction. Periodic evaluation meetings will be held with local stakeholders to assess challenges, identify best practices, and adjust the implementation process dynamically.

####### Sustainability and Scaling-Up Strategy

Based on the pilot results, a scaling-up plan will be developed to support expansion to other municipalities. The flexibility of the CP model will allow its replication in cities with distinct profiles, provided there is institutional commitment and minimum service capacity.

##### Monitor, Evaluate, and Adapt Outcomes: Sustain and Expand the CP

This stage corresponds to the KTA steps of monitoring knowledge use, evaluating outcomes, and sustaining knowledge application over time. Monitoring and evaluation activities will be carried out in municipalities participating in the pilot implementation and coordinated jointly with local health managers, STI program coordinators, and ESG. To ensure feasibility, the monitoring and evaluation plan is fully integrated into existing SUS information systems and municipal governance structures, requiring no new platforms or parallel data systems.

Each municipality will establish a CP monitoring committee composed of representatives from primary care, specialized STI services, psychosocial staff, urgent and emergency care professionals, and local management teams. This committee will review implementation progress, assess operational challenges, and guide ongoing adjustments. Monitoring will rely on routinely collected indicators derived from national health information systems, including Notifiable Diseases Information System-STI notifications (SINAN), Laboratory Test Control System (SISCEL), and Medication Logistics Control System-HIV care continuum (SICLOM), e-SUS APS (primary care records), the National Immunization Program Information System-vaccinations (SI-PNI), and Outpatient Information System of the Unified Health System (SIA/SUS) or Hospital Information System of the Unified Health System-service use (SIH/SUS). These sources provide real-time or periodic data on service performance and population outcomes without introducing additional burdens to municipal teams.

Key process indicators will include the number of rapid STI/HIV tests performed, referrals originated and completed, uptake of preexposure prophylaxis/postexposure prophylaxis, documented counseling sessions on harm reduction, and engagement with psychosocial services when indicated. Outcome indicators will focus on improved linkage to STI/HIV care, timeliness of treatment initiation, recurrent emergency visits for drug-related complications, and the accessibility of mental health care for individuals who engage in chemsex. App engagement metrics (eg, active users and most accessed content categories) will complement clinical indicators by assessing the reach and acceptability of digital educational tools.

Evaluation will follow a mixed methods approach, combining quantitative service data with qualitative feedback from municipal focal points and frontline teams. Regular virtual or in-person meetings will be held to discuss emerging barriers, identify service gaps, and refine workflows. Any required adjustments to the CP will be documented systematically to ensure traceability and to strengthen the adaptive capacity of the intervention.

Sustainability will be supported through continued collaboration with municipal managers and ESG leadership, incorporation of CP-related activities into routine municipal planning cycles, and dissemination of results through reports, scientific publications, and presentations in state-level governance forums such as Council of Municipal Health Secretariats, the State Health Secretariat technical committees, and intermunicipal planning meetings. At the end of the pilot period, consolidated findings will be presented to the State Health Secretariat to inform decisions regarding the feasibility of scaling the CP to additional municipalities. The final products, including the CP, recommendation guide, and mobile app, will be integrated into educational and academic programs to promote long-term capacity building and institutionalization. Through this iterative, data-driven process, the CP is expected to become a sustainable, evidence-informed component of comprehensive care for individuals who engage in chemsex within the HCN.

### Data Analysis

The quantitative analysis will include descriptive statistics to characterize participants and estimate the prevalence of chemsex, as well as logistic regression to identify associated factors (prevalence ratio), 95% CI; *P*<.05). Analyses will be conducted using SPSS (IBM Corporation) or Stata (Stata Corp) software.

Qualitative data from interviews and focus groups will be subjected to thematic analysis [31], supported by NVivo software (Lumivero). Triangulation will integrate quantitative and qualitative findings, along with results from the scoping review, to inform the development of the CP and educational materials.

During the implementation phase, process and outcome indicators will be monitored using official systems (SINAN, Mortality Information System, SIH/SUS) to evaluate the impact of the CP in pilot municipalities.

### Ethical Considerations

The study complies with resolution numbers 466/2012 and 510/2016 of the Brazilian National Health Council. Aligned with the Declaration of Helsinki, it was approved by the research ethics committee of the Ribeirão Preto College of Nursing from the University of São Paulo (85545824.2.0000.5393) and CRT-DST/AIDS-SP (85545824.2.3002.5375) in April 2025 and is set to begin in June 2025. All participants will be informed of the study’s objectives and will sign the ICF.

### Data Management Plan

Participants will sign the ICF, and data will be accessible only to the authorized research team, to be used exclusively for the purposes outlined in the study protocol. All information will be stored securely, with restricted access, and participants’ anonymity will be guaranteed throughout all phases of the research.

## Results

Participant recruitment and data collection began in June 2025 through a web-based platform and in-person strategies. By December 2025, a total of 3061 individuals had been screened online for eligibility and completion of the survey instruments.

In parallel, in-person recruitment and biological testing were conducted in health services and community-based settings. By December 2025, a total of 1723 participants had undergone rapid testing for STIs, including HIV, syphilis, and viral hepatitis.

Data collection is scheduled to be completed by June 2026. Data cleaning and preliminary descriptive analyses are scheduled for April-June 2026. Inferential analyses and qualitative data analysis are scheduled for July-September 2026. The development and validation of the Chemsex Care Pathway, including stakeholder workshops and logical model validation, are scheduled for October-December 2026. Dissemination of the main findings is expected in 2027.

## Discussion

We anticipate identifying higher chemsex prevalence among MSM than in other population segments, as well as polysubstance-use patterns that combine, in addition to the “classical” core described in European contexts (GHB/GBL, methamphetamine, and mephedrone/3-, 4-methylenedioxymethamphetamine), substances widely circulating in Brazil, such as cocaine/crack, ketamine, cannabinoids, and poppers, often associated with the adjuvant use of proerectile medications [5,7]. We further hypothesize associations between chemsex and greater vulnerability to sexual risk behaviors, worse well-being scores (WHO-5) and quality of life scores (EQ-5D-5L), as well as access barriers linked to stigma and perceived confidentiality [10,19]. Among managers and professionals, we expect heterogeneous CPs and low standardization of screening and management, training gaps (eg, toxicological emergencies related to GHB/GBL, counseling, and harm reduction), and incipient integration between surveillance and care, elements that should guide the development of a feasible, stigma-sensitive CP.

These expectations are aligned with recent literature describing heterogeneous chemsex prevalence among MSM in Brazil and emphasizing that substance use mixes depend on local markets and scenes, requiring that care responses be contextually adapted [5,7]. In parallel, global estimates indicate that SSU practices are not limited to MSM, with nonnegligible prevalence in the general population, expanding the scope of surveillance and planning [16]. In this sense, the hypothesis of more plural Brazilian patterns of polysubstance use is consistent with local availability and reports of differentiated risk profiles, for example, GHB/GBL intoxications and cardiovascular events associated with stimulants, which demand specific clinical routines and referral flows [10,19].

From an organizational standpoint, the architecture of Brazil’s HCN provides guidance for overcoming fragmentation and organizing thematic CPs with primary health care coordinating care [20]. The protocol’s planned collaboration with the ESG in São Paulo is expected to add territorial reach and operational feasibility to the analyses, enabling identification of bottlenecks across network points (primary health care–specialized care-mental health-emergency care), standardizing screening, and establishing protocols for acute events and for combined STI prevention. Findings from managers and professionals are expected to qualify, with service- and territory-level granularity, the “critical nodes” already suggested in reviews on chemsex interventions and harm reduction [19], strengthening the coproduction of care flows and educational materials.

The study presents methodological strengths that enhance the expected usefulness of the results. The sequential multimethod design combines a population survey, organizational diagnostics in services, and coproduction with stakeholders, allowing triangulation across prevalence/associated factors, installed capacity, and acceptability of CP components [5,19]. The target survey sample size was calculated to ensure precision in prevalence estimation and adequate power for comparisons among relevant subgroups, while the analytical plan using robust Poisson regression follows recommendations for estimating prevalence ratios in cross-sectional studies [40,41]. The projected events-per-variable adequacy supports stable, transportable models, adhering to contemporary criteria for events per variable and model planning [42-44].

Nevertheless, limitations must guide interpretation. Virtual sampling through snowball techniques and targeted campaigns may introduce selection biases (digital access and users of specific apps), requiring caution when generalizing findings. Self-reported data may introduce information bias for behaviors and substance use; data-quality procedures (mandatory fields, logic checks, and duplicate-response control) will mitigate, but not eliminate, this risk. The cross-sectional survey design does not permit causal inference between chemsex and well-being or quality-of-life outcomes; analyses will be interpreted as associations. In the managers/professionals module, convenience sampling limits population-level inferences; results should be understood as an organizational diagnostic useful for CP design and implementation.

Considering these strengths and limitations, potential implications for practice and policy include incorporating brief standardized screening at strategic HCN points; designing referral and counterreferral flows that integrate combined STI prevention, mental health, and harm reduction; establishing protocols for toxicological emergencies, particularly those related to GHB/GBL and stimulants, and implementing care-navigation strategies to reduce losses along the CP [19,20]. At a system level, findings may support state and municipal guidelines, multiprofessional training plans, and routine monitoring through process and outcome indicators, with a transversal emphasis on stigma reduction and equity.

Future directions include evaluating the CP from an implementation-science perspective, fidelity, acceptability, adoption, and cost, and measuring user outcomes such as testing coverage, linkage, and retention in care, and adverse events associated with substance use. Longitudinal studies could explore trajectories of use and mental health, as well as impacts on clinical outcomes (STIs, reinfections, and hospitalizations) and CP cost-effectiveness. Expansion to other states and nonurban contexts will test transferability and adaptation needs; digital interventions integrated into the CP, including peer education and just-in-time approaches, could be assessed in pragmatic trials.

The dissemination plan will include technical reports and policy briefs for SUS managers, educational materials and training packages for HCN teams, regional feedback sessions with ESG, academic outputs (preprints, articles, and conferences), and deposition of instruments and REDCap specifications in an open repository, as well as communication with civil society organizations in nonstigmatizing language. In summary, by combining population survey, organizational diagnostics, and coproduction, the protocol aims to inform and structure a feasible and acceptable chemsex CP within the SUS/HCN, enabling conclusions consistent with the proposed methodology, regarding the magnitude and associated factors of the phenomenon, organizational bottlenecks, and operational components of the CP, and creating a foundation for future effectiveness evaluations at scale [5,10,11,16,19,20,40-44].

## Appendix

Multimedia Appendix 1 Focus group topic guide.

Multimedia Appendix 2 Semistructured interview guide (English).

Multimedia Appendix 3 Peer review report from the National Council for Scientific and Technological Development (CNPq, Brazil).

## Abbreviations

CP: care pathway
CRT-DST/AIDS-SP: STD/AIDS Reference and Training Center of São Paulo state
ESG: epidemiological surveillance groups
GBL: gamma-butyrolactone
GHB: gamma-hydroxybutyrate
HCN: Health Care Network
ICF: informed consent form
KTA: Knowledge-to-Action
MSM: men who have sex with men
PRISMA-ScR: Preferred Reporting Items for Systematic Reviews and Meta-Analyses extension for Scoping Reviews
REDCap: Research Electronic Data Capture
SIA/SUS: Outpatient Information System of the Unified Health System
SICLOM: Medication Logistics Control System-HIV care continuum
SIH/SUS: Hospital Information System of the Unified Health System-service use
SINAN: Notifiable Diseases Information System-STI notifications
SI-PNI: National Immunization Program Information System-vaccinations
SISCEL: Laboratory Test Control System
SSU: sexualized substance use
STI: sexually transmitted infection
SUS: Unified Health System
WHO-5: World Health Organization-5 Well-Being Index
