# Supplementary material for: Development of a Line of Care for the Health of People Who Engage in Chemsex: Protocol for a Multimethod Study
Source: JMIR Res Protoc. 2026 Mar 26;15:e84068. doi: 10.2196/84068 (PMC13066781; doi:10.2196/84068)
Supplement: Multimedia Appendix 1 [file resprot_v15i1e84068_app1.docx]

# Appendix A – Focus Group Topic Guide

## Sociodemographic Information (Individual)

- Age

- Place of birth

- Nationality

- Race/skin color (self-reported): White, Black, Brown, Yellow, Indigenous

- Marital status: Single / Married / Divorced / Widowed / Other

- Do you have any disability? Yes / No

- If yes, which disability? Physical / Hearing / Visual / Intellectual

- Gender identity: cis man, trans man, transmasculine person, travesti, trans woman, cis woman, non-binary person, intersex, other

- Sexual orientation: heterosexual, homosexual, bisexual, pansexual, asexual, unsure, prefer not to answer, other

- Educational level

- State of residence

- City of residence

- Do you engage in Chemsex?

- Have you used psychoactive substances before or during sexual intercourse in the last 12 months?

- Are you a healthcare professional?

- Main professional role or position

- Type of health service where you work

- Have you been in this position for at least six months?

## Focus Group Guide – Managers and Health Professionals

- Have you heard of the term 'Chemsex' during your education or professional practice?

- Have you had any professional experience related to Chemsex?

- Have you ever assisted a patient who reported practicing Chemsex?

- Did you feel prepared to provide care related to Chemsex?

- Do you believe patients resist disclosing Chemsex practices? Why?

- Do you feel comfortable asking about sexual practices and substance use?

- Are there linguistic or cultural barriers in your service?

- Have you received training related to Chemsex care?

- What are the main physical and psychological risks associated with Chemsex?

- What services does your unit offer for people who practice Chemsex?

- Are there protocols or guidelines in your service to address Chemsex?

- Is there educational material available?

- Have you dealt with Chemsex-related emergencies?

- Does your service have protocols for substance-related emergencies?

- Does your service have protocols for Chemsex-related mental health issues?

- Does your service promote respect for sexual and gender diversity?

- Do you perceive inequalities in access to care for vulnerable populations?

- Does your service collect or monitor data on Chemsex?

- How do you assess coordination between levels of care?

- Do you consider Chemsex a priority in health policy planning?

- What strategies are essential to include Chemsex in SUS programs?

- Is there collaboration with NGOs or community groups?

- Is there stigma in the work environment regarding Chemsex?

## Focus Group Guide – People Who Practice Chemsex

- How would you describe your experience with Chemsex?

- How do you perceive the impact of Chemsex on your physical and mental health?

- Have you sought healthcare related to Chemsex?

- Do you feel comfortable discussing Chemsex with healthcare professionals?

- Have you experienced stigma or discrimination in health services?

- How do you evaluate the language used by health professionals?

- What changes would you suggest in healthcare services?

- What harm reduction strategies do you know or use?

- Do you think health professionals are prepared to assist people who practice Chemsex?

- What mental health support have you received or would you need?

- What recommendations would you make to improve care?

- Do you know NGOs or support groups related to Chemsex?
