# Supplementary material for: Development of a Line of Care for the Health of People Who Engage in Chemsex: Protocol for a Multimethod Study
Source: JMIR Res Protoc. 2026 Mar 26;15:e84068. doi: 10.2196/84068 (PMC13066781; doi:10.2196/84068)
Supplement: Multimedia Appendix 2 [file resprot_v15i1e84068_app2.docx]

# **Appendix B – Semi-Structured Interview Guide (English)**

This semi-structured interview guide is intended for individual interviews with health managers and healthcare professionals involved in the planning, management, or delivery of care within the Health Care Network (RAS). The interviews aim to support the validation of the Logical Model and the Line of Care for people who engage in Chemsex.

## Opening and Ethical Procedures

- Participant introduction and rapport building

- Explanation of the study objectives

- Confirmation of voluntary participation

- Reading and signing of the Informed Consent Form

- Permission for audio recording

- Explanation of confidentiality and anonymity

## Interview Questions – Managers and Health Professionals

- Can you describe your professional role and experience within the health system?

- What is your understanding of Chemsex and its relevance to public health?

- In your experience, how has Chemsex impacted healthcare services in your area?

- What are the main barriers faced by health services in addressing Chemsex-related health needs?

- What facilitators currently exist within the health system to support care for people who engage in Chemsex?

- How do you evaluate the preparedness of health professionals to address Chemsex-related issues?

- Are there existing protocols, guidelines, or care pathways in your service related to Chemsex or substance use in sexual contexts?

- How do stigma and discrimination affect access to care for people who practice Chemsex?

- How is coordination between primary care, specialized care, mental health services, and emergency care organized for this population?

- What gaps do you identify in the current organization of care for people who engage in Chemsex?

- What types of training or capacity-building initiatives would be necessary to improve care delivery?

- How do you assess the availability and adequacy of resources (human, financial, structural) to implement a Line of Care for Chemsex?

- In your opinion, should Chemsex be considered a priority within health policy planning? Why?

- What strategies would you recommend to integrate Chemsex-related care into existing SUS programs?

- How can intersectoral collaboration (e.g., NGOs, community organizations) contribute to improving care?

## Questions for Validation of the Logical Model and Line of Care

- Is the level of detail in the Logical Model sufficient to understand the necessary components and their interrelationships across the different levels of care?

- Is the logic of the proposed Line of Care complete, including all key elements required for implementation?

- Is the Logical Model theoretically sound? Do all elements fit together coherently?

- Are there alternative or additional pathways that could lead to the intended outcomes of the Line of Care?

- Have all relevant external contextual factors related to Chemsex and the health system been adequately identified and described in the model?

## Closing

Do you have any additional comments or recommendations that you believe are important for the development and implementation of the Line of Care for people who engage in Chemsex?
