# Supplementary material for: Development of a Line of Care for the Health of People Who Engage in Chemsex: Protocol for a Multimethod Study
Source: JMIR Res Protoc. 2026 Mar 26;15:e84068. doi: 10.2196/84068 (PMC13066781; doi:10.2196/84068)
Supplement: Multimedia Appendix 3 [file resprot_v15i1e84068_app3.pdf]

Resultado Final

Identificação da Proposta

**Número do Processo:**	405741/2024-3	**Solicitante:**	Ana Paula Morais Fernandes
**Chamada:**	ISTs 2024		
**Título do Projeto:**	?Pega-Ação?: Impacto do Chemsex nas Infecções Sexualmente Transmissíveis, saúde mental e sexual de homens que fazem sexo com homens e construção de uma linha de cuidado no contexto da política da Redução de Danos.		

Parecer de Deliberação Final

Nota Final

Nota	Ordem	Prioridade
8,17		P002

Resultado da Avaliação

Favorável

Justificativa:

"Proposta aprovada preliminarmente. A seguir análise pelo Comitê de Relevância Social visto que a Plataforma Carlos Chagas possui limitação para inserção de nova instância de julgamento conforme previsão desta Chamada: Esta proposta destaca-se por sua excepcional aplicabilidade, com alinhamento integral e profundo às diretrizes do Sistema Único de Saúde (SUS) e às políticas públicas nacionais e globais, como os Protocolos Clínicos e Diretrizes Terapêuticas (PCDTs), planos de eliminação e metas de controle para HIV/aids, hepatites virais e ISTs. A proposta facilita a concretização dos objetivos estabelecidos por essas políticas, contribuindo de maneira decisiva para a eliminação das infecções como problemas de saúde pública.A relevância da proposta é reforçada pela sua capacidade de gerar contribuições transformadoras, como a implementação de uma linha de cuidado integral para populações vulneráveis, a exemplo de pessoas envolvidas no Chemsex, uma prática abordada como foco no projeto ?Pega-Ação?.Essas intervenções oferecem novas perspectivas e práticas sustentáveis que fortalecem as capacidades institucionais e ampliam o acesso à saúde.A proposta não apenas responde às demandas emergentes, mas também estabelece paradigmas que poderão ser replicados em outras regiões do Brasil, contribuindo para a sustentabilidade e o impacto a longo prazo no SUS. Quanto ao impacto positivo nas condições de vida e saúde das comunidades e pessoas afetadas pelo HIV e aids, tuberculose, hepatites virais, ISTs ou micoses endêmicas, a proposta é marcada por pontos fortes excepcionais, como a implementação de uma linha de cuidado integral focada em populações vulneráveis, incluindo indivíduos que praticam Chemsex. Essa linha de cuidado, interligando serviços de saúde física e mental, assistência social e urgência/emergência, oferece suporte abrangente para necessidades biopsicossociais dessas populações, promovendo acesso facilitado a diagnósticos e tratamento precoce. A proposta é caracterizada por um impacto transformador, proporcionando mudanças paradigmáticas ao fortalecer a articulação entre serviços de saúde e equipamentos sociais. Ela responde diretamente às vulnerabilidades como estigma, discriminação, acesso limitado à saúde e riscos aumentados de transmissão de doenças, que afetam essas comunidades. Ainda assim, limitações relacionadas à infraestrutura e à capacitação regional em algumas áreas, especialmente fora dos grandes centros, demandam ajustes para otimizar a execução e maximizar os resultados almejados. Quanto ao plano para envolvimento e engajamento da sociedade civil e/ou representantes de pessoas e comunidades afetadas pelo HIV e aids, tuberculose, hepatites virais, ISTs ou micoses endêmicas, a proposta destaca-se por seu caráter excepcional e inovador. O plano é inovador ao integrar ONGs, lideranças comunitárias e redes digitais, como aplicativos de relacionamento, para facilitar o diálogo contínuo com as comunidades afetadas, especialmente HSH (homens que fazem sexo com homens) no contexto do Chemsex. Esse formato assegura um feedback constante, consultando as partes interessadas desde os estágios iniciais do projeto e adaptando estratégias com base em suas preocupações e necessidades, o que fortalece o alinhamento estratégico do projeto. Quanto ao número de doenças para as quais a proposta traria benefícios diretos, além das Infecções Sexualmente Transmissíveis (ISTs), nota-se que HIV/aids, ... (o restante do parecer poderá ser obtido junto ao email cobio@cnpq.br).Nota Critério A - 9; Nota Critério B - 9; Nota Critério C - 8; Nota Critério D - 7; Nota Critério E - 0; Nota Critério F - 0; Nota Ponderada CRS - 7,8. "

Recursos

Capital	Custeio	Bolsa	Valor Total
R$ 0,00	R$ 327.797,70	R$ 262.080,00	R$ 589.877,70

Data de Emissão

Data de Emissão do Parecer: 02/12/2024

Parecer de Deliberação final antes do período recursal

Critério: Mérito técnico-científico e caráter inovador

Peso: 3.0 **Nota:** 8.1

Critério: Adequação da metodologia aos objetivos do projeto, viabilidade técnica e gerenciamento de risco de execução.

Peso: 3.0 **Nota:** 7.8

Critério: Adequação da proposta à linha temática.

Peso: 1.0 **Nota:** 10.0

Critério: Infraestrutura da instituição, contrapartida para o projeto e experiência prévia do Coordenador e da sua equipe, considerando sua produção técnica e científica ou experiência profissional compatível com a realização do projeto

Peso: 1.0 **Nota:** 8.0

Critério: Adequação do cronograma de execução físico e financeiro

Peso: 1.0 **Nota:** 8.0

Critério: Plano de tradução e divulgação do conhecimento científico

Peso: 1.0 **Nota:** 8.0

Nota Final

Nota	Ordem	Prioridade

Resultado da Avaliação

Favorável

Justificativa:

"Proposta aprovada preliminarmente. A seguir análise pelo Comitê de Relevância Social visto que a Plataforma Carlos Chagas possui limitação para inserção de nova instância de julgamento conforme previsão desta Chamada: Esta proposta destaca-se por sua excepcional aplicabilidade, com alinhamento integral e profundo às diretrizes do Sistema Único de Saúde (SUS) e às políticas públicas nacionais e globais, como os Protocolos Clínicos e Diretrizes Terapêuticas (PCDTs), planos de eliminação e metas de controle para HIV/aids, hepatites virais e ISTs. A proposta facilita a concretização dos objetivos estabelecidos por essas políticas, contribuindo de maneira decisiva para a eliminação das infecções como problemas de saúde pública.A relevância da proposta é reforçada pela sua capacidade de gerar contribuições transformadoras, como a implementação de uma linha de cuidado integral para populações vulneráveis, a exemplo de pessoas envolvidas no Chemsex, uma prática abordada como foco no projeto ?Pega-Ação?.Essas intervenções oferecem novas perspectivas e práticas sustentáveis que fortalecem as capacidades institucionais e ampliam o acesso à saúde.A proposta não apenas responde às demandas emergentes, mas também estabelece paradigmas que poderão ser replicados em outras regiões

do Brasil, contribuindo para a sustentabilidade e o impacto a longo prazo no SUS. Quanto ao impacto positivo nas condições de vida e saúde das comunidades e pessoas afetadas pelo HIV e aids, tuberculose, hepatites virais, ISTs ou micoses endêmicas, a proposta é marcada por pontos fortes excepcionais, como a implementação de uma linha de cuidado integral focada em populações vulneráveis, incluindo indivíduos que praticam Chemsex. Essa linha de cuidado, interligando serviços de saúde física e mental, assistência social e urgência/emergência, oferece suporte abrangente para necessidades biopsicossociais dessas populações, promovendo acesso facilitado a diagnósticos e tratamento precoce. A proposta é caracterizada por um impacto transformador, proporcionando mudanças paradigmáticas ao fortalecer a articulação entre serviços de saúde e equipamentos sociais. Ela responde diretamente às vulnerabilidades como estigma, discriminação, acesso limitado à saúde e riscos aumentados de transmissão de doenças, que afetam essas comunidades. Ainda assim, limitações relacionadas à infraestrutura e à capacitação regional em algumas áreas, especialmente fora dos grandes centros, demandam ajustes para otimizar a execução e maximizar os resultados almejados. Quanto ao plano para envolvimento e engajamento da sociedade civil e/ou representantes de pessoas e comunidades afetadas pelo HIV e aids, tuberculose, hepatites virais, ISTs ou micoses endêmicas, a proposta destaca-se por seu caráter excepcional e inovador. O plano é inovador ao integrar ONGs, lideranças comunitárias e redes digitais, como aplicativos de relacionamento, para facilitar o diálogo contínuo com as comunidades afetadas, especialmente HSH (homens que fazem sexo com homens) no contexto do Chemsex. Esse formato assegura um feedback constante, consultando as partes interessadas desde os estágios iniciais do projeto e adaptando estratégias com base em suas preocupações e necessidades, o que fortalece o alinhamento estratégico do projeto. Quanto ao número de doenças para as quais a proposta traria benefícios diretos, além das Infecções Sexualmente Transmissíveis (ISTs), nota-se que HIV/aids, ... (o restante do parecer poderá ser obtido junto ao email cobio@cnpq.br).Nota Critério A - 9; Nota Critério B - 9; Nota Critério C - 8; Nota Critério D - 7; Nota Critério E - 0; Nota Critério F - 0; Nota Ponderada CRS - 7,8. "

Recursos

Capital	Custeio	Bolsa	Valor Total
R$ 0,00	R$ 327.797,70	R$ 262.080,00	R$ 589.877,70

Data de Emissão

Data de Emissão do Parecer: 31/10/2024

Parecer de Recomendação

Critério: Mérito técnico-científico e caráter inovador

Peso: 3.0 **Nota:** 8.1

Critério: Adequação da metodologia aos objetivos do projeto, viabilidade técnica e gerenciamento de risco de execução.

Peso: 3.0 **Nota:** 7.8

Critério: Adequação da proposta à linha temática.

Peso: 1.0 **Nota:** 10.0

Critério: Infraestrutura da instituição, contrapartida para o projeto e experiência prévia do Coordenador e da sua equipe, considerando sua produção técnica e científica ou experiência profissional compatível com a realização do projeto

Peso: 1.0 **Nota:** 8.0

Critério: Adequação do cronograma de execução físico e financeiro

Peso: 1.0 **Nota:** 8.0

Critério: Plano de tradução e divulgação do conhecimento científico

Peso: 1.0 **Nota:** 8.0

Nota Final

Nota	Ordem	Prioridade

Resultado da Avaliação

Recomendada

Justificativa:

Este estudo pretende analisar os impactos do Chemsex nas Infecções Sexualmente Transmissíveis, saúde mental e sexual de homens que fazem sexo com homens para a construção de uma linha de cuidado no contexto da política de Redução de Danos. A proponente apresenta contribuições para sua área de expertise, bem como a equipe inserida na proposta é constituída por pesquisadores de excelência. Contudo, a proposta apresenta problemas metodológicos e pouco caráter inovador. O cronograma está adequado para avaliar a execução das atividades da proposta. Assim, este Comitê avaliou comparativamente os múltiplos aspectos das propostas apresentadas, incluindo mérito científico e de inovação, a produção técnico-científica da coordenadora e equipe, a liderança do grupo e, também, considerou a análise externa dos pareceristas ad hoc. Diante do exposto, o Comitê avaliador RECOMENDA o apoio à proposta, ficando este condicionado à disponibilidade orçamentária.

Recursos

Capital	Custeio	Bolsa	Valor Total
R$ 0,00	R$ 327.797,70	R$ 262.080,00	R$ 589.877,70

Data de Emissão

Data de Emissão do Parecer: 14/10/2024

Parecer de Pré-seleção

Critério: O proponente atende ao item 3.2.1 da Chamada?

SIM

Critério: A instituição de execução do projeto é uma Instituição Científica, Tecnológica e de Inovação (ICT) cadastrada no Diretório de Instituições do CNPq?

SIM

Critério: A proposta apresenta projeto anexo à Chamada com, no máximo, 12 páginas?

SIM

Nota Final

Nota	Ordem	Prioridade

Resultado da Avaliação

Enquadrada

Justificativa:

A proposta atende as exigências da chamada.

Data de Emissão

Data de Emissão do Parecer: 25/09/2024

Parecer de Ad Hoc

Critério: Mérito técnico-científico e caráter inovador da proposta.

Projeto que tematiza questão relevante, mas o faz de modo pouco cuidadoso, com frases, por vezes desconexas, como: "O ciclo de criação do conhecimento com compõe" [sic] e circulares, c durante a atividade sexual, está associado ao aumento de [...] e transtornos mentais, como dependência química [verbatim, sic]. Obviamente, uma fração das pessoas que praticam o chemse de como pessoas que já são dependentes químicos viriam a praticar o chemsex, assim como se a prática de chemsex viria, supostamente, a agravar a dependência química. Uma fração substa associação necessária (ou sequer factível) com chemsex. Pelo contrário, determinados padrões de uso, como de opioides de alta potência, como o fentanil, e o recentemente identificado Nitaz (https://www.sciencedirect.com/science/article/pii/S2665910724000240) induzem de forma profunda e rápida depressão rápida, o que impede a prática sexual, frequentemente estão asso mil óbitos anuais nos EUA em anos recentes). Aparentemente, a falta de familiaridade da proponente principal e sua equipe com a questão específica do uso de substâncias e dos riscos e danc mesmo nas suas cinco páginas de texto, mas não contenha nenhuma descrição, ainda que sumária, das substâncias a serem pesquisadas e nenhuma menção à extensa bibliografia recente sol

Critério: Adequação da metodologia aos objetivos do projeto, viabilidade técnica e gerenciamento de risco de execução.

Há diversos erros na formulação da proposta do ponto de vista metodológico, mas nos limitaremos a indicar os mais relevantes: 1) Não há qualquer explicação para o primeiro objetivo, que milhões de pessoas, o que em se tratando de uma prática privada, e, em diversos contextos, estigmatizada, faz com que, literalmente, a missão seja a de "procurar uma agulha no palheiro". E brilhante conjunto de métodos, aplicados a uma imensa cidade, Chicago, seja o da equipe de Sampson (ver resumo em seu magistral livro: https://press.uchicago.edu/ucp/books/book/chica prevalências as mais diversas, mas em nenhum momento há uma definição, básica que seja, a qualquer amostra. Simplesmente, não existe plano amostral! Portanto, ainda que seja possível o calcular taxas! 3) Menciona-se inferência estatística, mas sem qualquer esclarecimento de que amostras probabilísticas constituem o padrão ouro da inferência estatística. Obviamente, não ha análise, apostando-se, portanto, em amostras não probabilísticas. Mas, na ausência absoluta de um plano amostral, não é possível pensar em métodos de aprimoramento da inferência estatíst https://projecteuclid.org/journals/statistical-science/volume-32/issue-2/Inference-for-Nonprobability-Samples/10.1214/16-STS598.full). 4) Menciona-se a utilização de instrumentos valid respeito de que instrumentos seriam esses. 5) As normas mais básicas de revisões de escopo estão sistematizadas em: https://www.prisma-statement.org/scoping, mas não há nenhuma cor como: "Um relato narrativo [sic] dos resultados será criado com base em categorias temáticas" (verbatim). Todo relato é narrativo, ou, na pós-modernidade, por vezes, fragmentário e lacuna escopo. Revisões de escopo são, necessariamente, compatíveis com os conceitos fundacionais de Walter Benjamin sobre a função do narrador, texto amplamente disponível em tradução brasi tradução de SP Rouanet) e hoje disponível inclusive no Youtube (https://www.youtube.com/watch?v=7Zj2a-4h-VM). 6) Bardin produziu seu notável trabalho na década de 1970. Transcorrid (e.g. https://ibpad.com.br/ciencia-dados/no-brasil-analise-de-conteudo-e-sinonimo-de-bardin-por-que-isso-e-um-problema/), assim como alternativas bastante mais avançadas que incorpo textuais. Uma vez que propõe-se um investimento maciço em softwares, o que será comentado adiante, por que não lançar mão de métodos avançados de análise linguística, que são não aper (http://corpora.lancs.ac.uk/lancsbox/index.php). Há diversos outros erros, de menor monta, comentados mais adiante.

Critério: Adequação da proposta à linha temática.

Este Não é o problema da proposta, mas sim seus erros de formulação.

Critério: Infraestrutura da instituição, contrapartida para o projeto e experiência prévia do Coordenador e da sua equipe, considerando sua produção técnica e científica ou experiência profis

A infraestrutura é adequada, mas nenhum dos proponentes, a começar pela coordenadora, demonstra qualquer familiaridade com a questão do uso de substâncias em si. Percorri seu CV Latt indexadas, e alguns artigos em revistas indexadas, com um impacto ora muito modesto, ora mais relevante. O impacto agregado das suas publicações é muito baixo, o que seria de se esperar impacto. A sistematização quantitativa dessas afirmações está disponível em Wang e Barabási, livro que os autores deveriam ler, visando seu aprimoramento. Eu o fiz e, por isso mesmo, reco https://www.cambridge.org/core/books/science-of-science/572A745A6F97B55A263F5E86225E3F70

Critério: Adequação do cronograma de execução físico e financeiro.

Como alocar recursos a algo tão mal definido, como mapear cenas na capital do Estado de São Paulo e interior? (sem qualquer explicação adicional) A leitura do livro de Sampson ensinaria a desafios são equacionáveis, mediante uma combinação inteligente de observação etnográfica, análise de redes sociais, gravações de espaços públicos, como cenas de tráfico, e a utilização (a recursos computacionais avançados, por exemplo, integrando imagens geradas pelo Google Street View e o R. Há uma subutilização surpreendente das bibliotecas do R, que está citado de for ambiente computacional, que comporta um número potencialmente infinito de bibliotecas que servem às mais diversas finalidades. Muito provavelmente, os autores misturaram o R em si com aquisição de diversos softwares que apenas duplicam as bibliotecas do R, só que com muito menor funcionalidade e flexibilidade. Começando pelo começo, uma definição correta das coisas se forma brilhante Wittgenstein. Comecemos, portanto, pela citação oficial do próprio R (disponível em: https://ropensci.org/blog/2021/11/16/how-to-cite-r-and-r-packages/). R Core Team (2 Foundation for Statistical Computing, Vienna, Austria. URL https://www.R-project.org/. Tomando como exemplo um dos pacotes a ele integrados: Jeroen Ooms (2021). magick: Advanced Gra https://CRAN.R-project.org/package=magick

Critério: Plano de tradução e divulgação do conhecimento científico

O plano em si é bem formulado, mas traduz conhecimentos bastante imprecisos, como o referente a taxas para as quais não existem denominadores. Gestores públicos tomam decisões em c fundamental reduzir essa margem de incerteza e subsidiar as decisões mediantes achados empíricos robustos. Nesse sentido, métodos deficientes não permitem reduzir as margens de incert auxiliam o cidadão a acompanhar e debater as políticas públicas. A referência que segue sendo clássica é a do Prêmio Nobel Daniel Kahnneman e seus colaboradores do mais alto nível. Esta re autores: https://www.amazon.co.uk/Judgment-under-Uncertainty-Heuristics-Biases/dp/0521284147/ref=sr_1_5?crid=2R7ICUI7QIVL&dib=eyJ2IjoiMSJ9.7JvrPd-aFHZ_6fCZ8FWv0BXv-PkY RjY2yG2FlCkNHmPCXbdfC2niIoK1BYnsDBgMMIrtrqSHk1dlwdkS4Tha8i7_8278RK1w4jW7PfqPOpif-sLgGIbseKmyDQrcrf3x9dTNI1msMOHEmmPcGjcY-T2dZxPR3u6TsQXNKFiserrgFVZL2JWw8ofxiQLdOtPJbXMP5tpebitDKyHeZlZAQsHhJSRYRw.D5JR_6fkoQl_fNb2HSjugojzvnB1qIWCmbTQu9yjGAU&dib_tag=se&keywords=amos+tversky&qid=17 5

Nota Final

Nota	Ordem

Resultado da Avaliação

Não Recomendado

Justificativa:

Como acima exposto, o problema é de inegável relevância e a interação com gestores e profissionais de saúde é extremamente louvável, mas tudo está apoiado em uma metodologia pouco integrada, redigida de f linguística, sociologia, epidemiologia, etc.) nos últimos 50 anos, o que ocorre em diversas ocasiões.

Data de Emissão

Data de Emissão do Parecer: 06/09/2024

Parecer de Ad Hoc

Critério: Mérito técnico-científico e caráter inovador da proposta.

A presente proposta apresenta mérito científico ao propor uma abordagem inédita de analisar os impactos do Chemsex nas Infecções Sexualmente Transmissíveis, saúde mental e sexual de homens que fazem sexo com homens para a construção de uma linha de cuidado no contexto da política de Redução de Danos, um grave problema de impacto na disseminação de ISTs. Além do mapeamento do problema, a proposta visa desenvolver tecnologias educacionais e estratégias de educação em saúde, construir, implantar, monitorar e avaliar, de forma piloto e com as parcerias estabelecidas no projeto, uma linha de cuidado para atenção à saúde de pessoas que praticam Chemsex no contexto da política da Redução de Danos.

Critério: Adequação da metodologia aos objetivos do projeto, viabilidade técnica e gerenciamento de risco de execução.

A pesar da importância e do mérito científico apresentados na justificativa do projeto, no que se refere a metodologia, não está claro qual o n amostral a ser avaliado de pessoas que fazem uso do Chemsex, assim como não há descrição da metodologia a ser empregada para o alcance do objetivo no. 3 que propõe avaliar a prevalência de ISTs/HIV/Aids, hepatites virais, sífilis e Mpox. Sem uma abordagem metodológica de avaliação da ocorrência de infecção por esses agentes é impossível estimar a prevalência das mesmas.Quais métodos laboratoriais serão usados para análise de investigação das possíveis infecções? Sorologia? Biologia Molecular? A ausência desta definição é um ponto que compromete a qualidade do projeto. Ademais, se a proposta visa avaliar o impacto da Chemsex nas ISTs seria importante haver um grupo controle de não usuários para efeito de comparação.

Critério: Adequação da proposta à linha temática.

A proposta é adequada a linha temática ao propor um tema relacionado diretamente a ações de vigilância e prevenção para eliminação do HIV/Aids, tuberculose e ISts, hepatites virais, contudo como comentado acima sua metodologia traz falha na descrição de como o projeto pretende alcançar o propósito de vigilância sem averiguar a real prevalência no grupo populacional escolhido para o estudo.

Critério: Infraestrutura da instituição, contrapartida para o projeto e experiência prévia do Coordenador e da sua equipe, considerando sua produção técnica e científica ou experiência profissional compatível com a realização do projeto

Há uma breve descrição da infraestrutura de apoio a realização do projeto que aparentemente, a julgar pelas instituições envolvidas, parece ser adequada para o apoio a realização da proposta. A despeito da ausência de informação de infraestrutura para as análises de investigação da prevalência das ISTs.

Critério: Adequação do cronograma de execução físico e financeiro.

O cronograma encontra-se adequado ao prazo estabelecido no edital.

Critério: Plano de tradução e divulgação do conhecimento científico

A proposta apresenta como estratégia de tradução e divulgação do conheciemtno a produção de materiais instrucionais como, aplicativo digital, e-book, guias de recomendações e letramento, cartilhas, infográficos, animas e vídeos, visando apoiar treinamentos e a execução de protocolos no contexto do Chemsex, os quais também serão distribuídos nos serviços de saúde e em plataformas digitais, de modo a facilitar o acesso contínuo para profissionais de saúde e a população em geral. Neste quesito a proposta é bem fundamentada.

Nota Final

Nota	Ordem	Prioridade

Resultado da Avaliação

Recomendado

Justificativa:

A proposta é meritória na sua concepção ao abordar uma questão importante no contexto da saúde de homens que fazem sexo com homens. O escopo do projeto está centrado na avaliação do impacto do Chemsex nas ISTs/HIV/Aids, sífilis, hepatites virais e Mpox, assim como na saúde mental e sexual dos usuários. Embora a proposta tenha seu mérito científico indiscutível, há problemas metodológicos que não permitem o alcance do objetivo no. 3 que, explicitamente, diz que será avaliada a prevalência de ISTs/HIV/Aids, sífilis, hepatites virais e Mpox nos praticantes de Chemsex. A não definição de qual metodologia será usada para determinar a prevalência dessas infecções é um gap que prejudica a avaliação da proposta neste quesito. Assim, sendo, julgo que a proposta poderia ser recomendada a depender da análise comparativa com conjunta as demais propostas submetidas a Chamada 34/2024.

Data de Emissão

Data de Emissão do Parecer: 03/09/2024

Voltar
